# Effectiveness of Menthacarin on symptoms of irritable bowel syndrome

**DOI:** 10.1007/s10354-018-0635-1

**Published:** 2018-05-04

**Authors:** Ahmed Madisch, Stephan Miehlke, Joachim Labenz, Berenike Stracke, Stephan Köhler

**Affiliations:** 1 Medical Department I, Academic Teaching Hospital Siloah, Stadionbrücke 4, 30459 Hannover, Germany; 2 Center for Digestive Diseases, Internal Medicine Center Eppendorf, Hamburg, Germany; 3 Medical Department, Diakonie Klinikum Jung-Stilling, Siegen, Germany; 4 Clinical Research Department, Dr. Willmar Schwabe GmbH & Co. KG, Karlsruhe, Germany

**Keywords:** Peppermint oil, Caraway oil, Irritable bowel syndrome, Menthacarin, Functional dyspepsia, Pfefferminzöl, Kümmelöl, Reizdarmsyndrom, Menthacarin, Funktionelle Dyspepsie

## Abstract

Irritable bowel syndrome (IBS) and functional dyspepsia (FD) are common functional gastrointestinal disorders with overlapping symptoms. Effectiveness and safety of Menthacarin (Menthacarin® is the active ingredient of the product Carmenthin® [Dr. Willmar Schwabe GmbH & Co. KG, Karlsruhe, Germany]) in FD treatment were already demonstrated. We assessed the effectiveness of Menthacarin in reducing concomitant IBS-associated symptoms in FD patients. A systematic search to identify eligible double-blind, randomized controlled trials (RCTs) investigating Menthacarin in FD patients and focusing on IBS-associated symptoms was performed. Three out of five identified RCTs included a total of 111 eligible subjects, which allowed for summary statistics and inclusion into subgroup analysis for FD patients with IBS-associated symptoms. With pain intensity values decreasing by 50–75% on average during 28 days of treatment in patients with accompanying IBS, the subgroup analysis indicates beneficial treatment effects of Menthacarin that are similar to those found for FD patients in the primary analyses. The reduction of IBS-associated symptoms in FD patients suggests Menthacarin as a treatment option for IBS patients.

## Zusammenfassung

Das Reizdarmsyndrom (RDS) und die funktionelle Dyspepsie (FD) sind verbreitete funktionelle gastrointestinale Beschwerden mit überlappenden Symptomen. Wirksamkeit und Sicherheit von Menthacarin (Menthacarin® ist der Wirkstoff des Produkts Carmenthin® [Dr. Willmar Schwabe GmbH & Co. KG, Karlsruhe, Germany]) wurden in der Behandlung der FD bereits gezeigt. Gegenstand der vorliegenden Untersuchung war die Wirksamkeit von Menthacarin in der Behandlung IBS-typischer Begleitsymptome bei FD-Patienten. Zur Auffindung geeigneter doppelblinder randomisierter kontrollierter Studien (RCT) über die Anwendung von Menthacarin bei FD-Patienten mit Fokus auf das Auftreten IBS-typischer Symptome wurde eine systematische Literatursuche durchgeführt. Dabei fanden sich 5 entsprechende RCT. In 3 davon wurde eine Gruppe von insgesamt 111 geeigneten Patienten beschrieben. Die daraus gewonnenen Daten ermöglichten die Erstellung zusammenfassender Statistiken und die Durchführung einer Subgruppenanalyse von FD-Patienten mit begleitenden IBS-Symptomen. Mit einer durchschnittlichen Reduzierung der Schmerzintensität von 50–75 % bei den FD-Patienten mit IBS-Symptomen nach 28 Tagen zeigt die Subgruppenanalyse günstige, mit den Ergebnissen der Primäranalyse der FD-Patienten vergleichbare Behandlungseffekte von Menthacarin. Der Rückgang IBS-typischer Begleitsymptome bei FD-Patienten deutet auf Menthacarin als mögliche Therapieoption für IBS-Patienten hin.

## Introduction

In Western industrialized countries, the disease prevalence reported for irritable bowel syndrome (IBS) is around 15% in the adult population (e. g., [1, 2]) and more common in women [3]. Half of all new patients in general practice suffer from functional disorders, and most IBS patients who seek medical care are managed by general practitioners (GPs; [4]).

IBS is characterized by recurrent abdominal pain or flatulence usually accompanied by altered bowel habits, and is commonly associated with features of disordered defecation [5, 6]. In patients seeking treatment for IBS, repeated consultations with extensive diagnostic work-up, expenses for medication, and time lost from work place represent a significant burden for the health system [7].

Although the pathophysiology of IBS is not yet completely understood, recent research suggests that local immune activation and altered barrier function, such as increased intestinal mucosa permeability, and reflex motor responses may contribute to the development of the disease [8]. Moreover, the de novo development of IBS following an episode of bacterial gastroenteritis indicates a potential involvement of a bacterial pathogen in the etiology of the condition [9].

The symptoms of IBS and functional dyspepsia (FD) were found to be frequently overlapping (e. g., [10]). Talley [11] estimated that at least one third of patients with FD also suffer from IBS. Considering the beneficial effects of peppermint oil in IBS, the established efficacy of a proprietary combination of peppermint oil and caraway oil (Menthacarin^1^) in FD [12], and the overlap of symptoms of the two syndromes, the administration of Menthacarin to patients with IBS could be a therapeutic option. However, no clinical trials have been conducted to date to investigate the efficacy of the herbal combination Menthacarin in IBS specifically. To evaluate the effect of Menthacarin on IBS symptoms, all randomized, double-blind trials conducted with Menthacarin in patients with FD were identified and data were re-analyzed for the subgroup of those patients additionally suffering from IBS.

## Materials and methods

A systematic literature search was conducted using MEDLINE and Google Scholar (search terms: “peppermint oil” and “caraway oil” and “dyspepsia”) in order to identify all double-blind clinical trials with the proprietary combination of peppermint oil and caraway oil (hereinafter referred to as Menthacarin) in patients with FD published until January 2018. Additionally, the reference lists of these articles were searched for further relevant studies. Five double-blind randomized controlled trials (RCTs) with a randomized treatment duration of 28 days were identified [13–17]. All study protocols were approved by the appropriate ethics committees. All patients gave their written informed consent prior to their inclusion in the respective study.

To allow a meaningful computation of summary statistics, trials were included in the analysis if at least five patients in the Menthacarin arm of the study had concomitant IBS. This was fulfilled by three trials ([13, 15, 17]; basic characteristics are shown in Table 1).

**Table 1:** Characteristics of trials included in the analysis

	Trial A	Trial B	Trial C
Publication	May et al. [17]	Freise and Köhler [13]	Madisch et al. [15]
Treatment	3 × 1 enteric-coated capsule/day Menthacarin (total daily dose of 270 mg peppermint oil and 150 mg caraway oil) or placebo for 4 weeks	3 × 1 enteric-coated capsule/day Menthacarin (total daily dose of 270 mg peppermint oil and 150 mg caraway oil) or enteric-soluble comparator for 4 weeks (total daily dose of 108 mg peppermint oil and 60 mg caraway oil)	2 × 1 enteric-coated capsule/day Menthacarin (total daily dose of 180 mg peppermint oil and 100 mg caraway oil) or 3 × 10 mg cisapride for 4 weeks
Patients with IBS/total (ITT)	Menthacarin: 12/22 (55%)	Menthacarin: 40/108 (37%)	Menthacarin: 5/60 (8%)
	Placebo: 10^a^/23 (44%)	Comparator: 42/105 (40%)	Comparator: 3/58 (5%)
Primary selection criteria	Adult outpatients suffering from FD, with or without concomitant IBS symptoms; presence of at least two dyspeptic and/or bowel associated symptoms for at least 14 days; absence of an organic cause of symptoms; informed consent prior to study participation		
Primary outcome measure	Change in intensity of pain	Change in intensity of pain	Change in intensity of pain
	CGI Item 2		
Secondary outcome measures with relevance to IBS	Flatulence; feelings of pressure/heaviness/fullness; diarrhea	Flatulence; feelings of pressure/heaviness/fullness; diarrhea	Flatulence; feelings of pressure/heaviness/fullness; diarrhea
	CGI items 1 + 3	CGI items 1–3	CGI items 1–3

Menthacarin® is the active ingredient of the product Carmenthin® (Dr. Willmar Schwabe GmbH & Co. KG, Karlsruhe, Germany)

*IBS* irritable bowel syndrome, *FD* functional dyspepsia, *ITT* intention-to-treat, *CGI* clinical global impressions scale

^a^One patient out of 10 was not evaluable because of missing data under therapy

In all three trials, Menthacarin was given as enteric-coated capsules. The primary outcome measure for treatment efficacy was the change in the intensity of epigastric pain between the start of the double-blind phase and treatment end. In Trial A, the intensity of epigastric pain was rated by the patient on a six-point scale ranging from 0 (“no pain”) to 5 (“extremely severe pain”). All other trials used a 10-cm visual analogue scale (0 = absent and 10 = maximum intensity). Secondary outcome measures of treatment efficacy included self-ratings of other cardinal symptoms of FD and IBS (pain frequency; feelings of pressure, heaviness, tension, or fullness; intolerance to food; nausea; flatulence; irregular stools), and the Clinical Global Impressions scale (CGI; [18]) as an investigator’s rating (for secondary outcome measures with particular relevance to IBS, see Table 1). Other secondary efficacy measures and safety assessments performed during the trials, as well as further details regarding the trial procedures, are described elsewhere [13, 15, 17]. Whereas Trial A was conducted to demonstrate superiority in efficacy of Menthacarin over placebo, Trial B aimed at demonstrating non-inferiority of Menthacarin given as enteric-coated capsules in comparison to an immediate-release (enteric-soluble) formulation containing the same essential oils, and Trial C demonstrated non-inferiority of Menthacarin in comparison to the propulsive agent cisapride.

### Subgroup analyses

The subgroups defined for our investigation included all patients who were eligible for the analysis of treatment effectiveness according to the original intention-to-treat (ITT) analysis sets of the five primary studies and who suffered from IBS in addition to FD. The diagnosis of IBS was based on the investigators’ clinical assessment. The data of the patients with concomitant IBS were re-analyzed and compared with the results of all patients with FD.

### Statistical analyses

The review includes descriptive statistics (i. e., mean and standard deviation) for the change in intensity of epigastric pain between the beginning and end of randomized treatment, separately performed for patients with FD and for the patients with concomitant IBS. In addition, we include a detailed presentation of the change in the CGI severity of illness rating (item 1) and of other outcome measures that describe symptoms associated with IBS (e. g., feelings of pressure, heaviness, or fullness; flatulence, diarrhea) and that were assessed in all trials. For the changes in the intensity of pain rating and in CGI item 1, the ratio of the mean treatment changes between the Menthacarin group and the respective comparator group were calculated and the associated Fieller 95% confidence intervals (CIs) with corresponding *p*-values [19] were determined for both FD patients with and without concomitant IBS symptoms. Due to the descriptive nature of the analyses, the *p*-values were regarded as significant for *p* < 0.05 and were not adjusted for multiple testing. For the change in intensity of pain and the CGI item 1, the last observation carried forward method was applied to impute missing data of patients withdrawn prematurely. The analyses were done with SAS version 9.2.

## Results

### Summary of primary analyses

In the primary analyses of all patients (with or without concomitant IBS symptoms), Menthacarin was significantly superior to placebo in reducing the pain associated with FD in Trial A (*p* < 0.05) after 4 weeks of randomized treatment (two-sided tests). In Trials B and C, Menthacarin given as an enteric-coated formulation was determined not to be inferior to an enteric-soluble formulation containing the same essential oils (*p* < 0.01; non-inferiority margin ∆ = 10%) and not to be inferior to cisapride (*p* < 0.05; ∆ = 1 cm on the 10 cm VAS; two-sided tests). Further details are reported in the primary publications [13, 15, 17].

### Subgroup analysis

In Trials A, B; and C, 111/376 (30%) patients evaluable for efficacy suffered from concomitant IBS symptoms, and the within-group percentage of patients with concomitant IBS symptoms varied between 5% (Trial C, cisapride) and 55% (Trial A, Menthacarin; Table 1).

### Intensity of epigastric pain

Table 2 presents a comparison between the average change in epigastric pain ratings in patients randomized to Menthacarin and to the comparator group. The analyses were performed separately for patients with concomitant IBS symptoms and for all patients assessed for efficacy. In Trials A and B, patients with or without concomitant IBS symptoms showed comparable decreases in intensity of pain in the Menthacarin group. For the IBS subset in Trial A, this corresponded to a reduction in intensity of pain which was nearly twice as large in the Menthacarin group as in the placebo group (ratio of means, Menthacarin/placebo: 1.98, 95% CI: 0.92–12.00). This effect showed a trend for an advantage of Menthacarin over placebo (*p* = 0.079), but was not significant due to the limited number of patients in the study. In Trial C, the average reduction in intensity of pain in patients with concomitant IBS symptoms was somewhat less pronounced than in the complete dataset, but again this applied to both treatment groups, and the small number of patients with IBS in this trial (5 and 3 for Menthacarin and cisapride, respectively) has to be taken into consideration.

**Table 2:** Intensity of pain – change during double-blind treatment (mean ± SD, or ratio of means and 95% CI)

		Total dataset	Patients with IBS
Trial A^a^	Menthacarin	−2.47 ± 1.31 (*n* = 19)	−2.42 ± 1.24 (*n* = 12)
	Placebo	−1.35 ± 1.66 (*n* = 20)	−1.22 ± 1.72 (*n* = 9)
	Ratio of means	1.83 [1.08, 3.83]	1.98 [0.92, 12.00]
Trial B^b^	Menthacarin	−3.59 ± 2.11 (*n* = 108)	−3.51 ± 2.01 (*n* = 40)
	Enteric-soluble formulation	−3.30 ± 2.04 (*n* = 105)	−3.52 ± 1.80 (*n* = 42)
	Ratio of means	1.09 [0.93, 1.29]	1.00 [0.78, 1.27]
Trial C^b^	Menthacarin	−4.57 ± 2.80 (*n* = 60)	−3.62 ± 3.40 (*n* = 5)
	Cisapride	−4.59 ± 2.48 (*n* = 58)	−3.97 ± 2.75 (*n* = 3)
	Ratio of means	1.00 [0.80, 1.23]	0.91 [–∞, +∞]

Menthacarin® is the active ingredient of the product Carmenthin® (Dr. Willmar Schwabe GmbH & Co. KG, Karlsruhe, Germany)

*SD* standard deviation, *CI* confidence interval, *IBS* irritable bowel syndrome

^a^Scale: 0 … 5

^b^Scale: 0 … 10

The ratio of the mean treatment changes can be taken as an indicator for the effect of Menthacarin relative to the comparator. As seen with the absolute changes versus baseline, only negligible differences were observed between the ratios in the total dataset and in the IBS subset (Table 2). The data thus indicate that the presence of concomitant IBS symptoms did not relevantly modify the relative effect of Menthacarin in reducing intensity of pain.

While the reduction of the baseline intensity of pain was 38% for placebo after 4 weeks in Trial A, the reduction for Menthacarin was considerably higher (Trial A: 75%, Trial B: 58%, Trial C: 50%). In Trials A and B, about 90% of the patients in the total dataset as well as in the IBS subset, who received Menthacarin or the enteric-soluble comparator, had lower intensity of pain ratings at treatment end than at baseline. On the other hand, the percentage of patients with pain alleviation in the placebo group of Trial A did not exceed 45% for the total dataset and the IBS subset alike (due to the small number of patients with IBS, such an analysis was not done for Trial C).

### IBS-associated symptoms

**Fig. 1:**
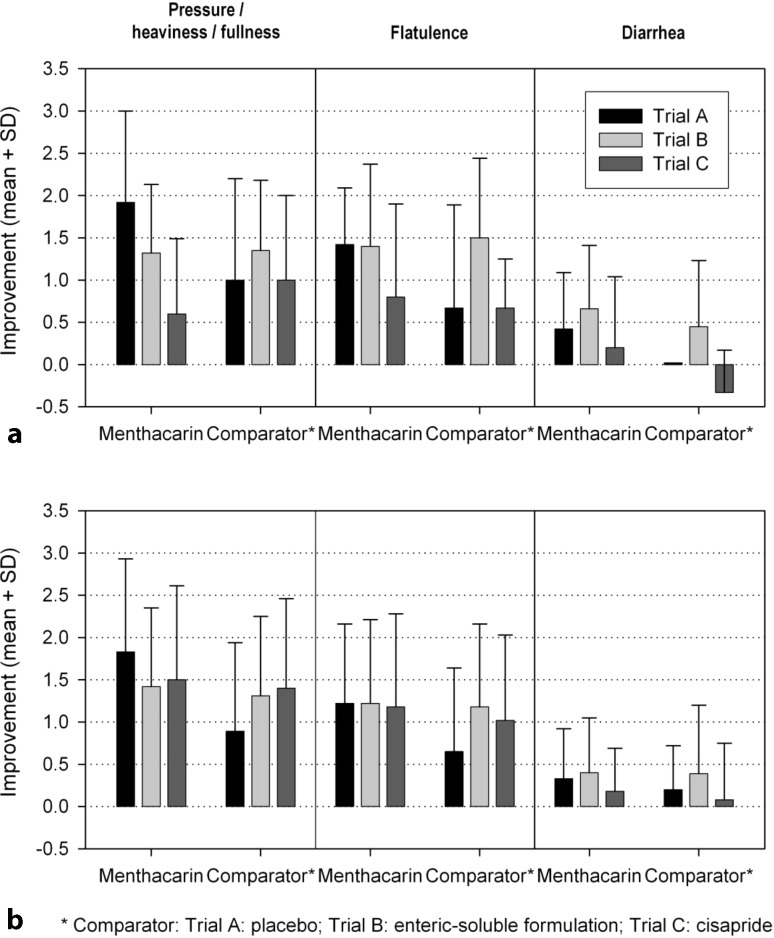
Improvement of IBS-associated symptoms during double-blind treatment in patients with concomitant IBS symptoms (chart A) and in the total dataset (chart B). *SD* standard deviation, *IBS* irritable bowel syndrome. Menthacarin® is the active ingredient of the product Carmenthin® (Dr. Willmar Schwabe GmbH & Co. KG, Karlsruhe, Germany)

In addition, Trials A through C assessed the presence and intensity of flatulence, diarrhea, and feelings of pressure, heaviness, tension, or fullness on four-point self-rating scales. All trials showed an average reduction in the intensity of symptoms versus baseline for Menthacarin (Fig. 1). Menthacarin was more effective than placebo (Trial A) and the reduction of the concomitant IBS symptoms pressure/heaviness/fullness, flatulence, or diarrhea in the Menthacarin group were about twice as large as in the placebo group. Furthermore, Menthacarin given as enteric-coated capsules showed no relevant differences to the immediate-release formulation containing the same ethereal oils (Trial B) or to cisapride (Trial C). This applied to the complete datasets as well as to the IBS subsets for which similar results were observed.

### Clinical global impressions

The changes in the investigators’ ratings of severity of disease (CGI item 1) are summarized in Table 3. In Trial A, the treatment groups’ means for change during double-blind treatment as well as their ratio were comparable between the total dataset and the IBS subset. In trial B, the patients with IBS treated with Menthacarin showed a slightly lower average improvement than the total dataset, while those receiving the enteric-soluble formulation had a slightly higher mean value. Since these differences pointed in opposite directions, the resulting ratio of the means also showed a deviation from the ratio obtained for the total dataset. In Trial C, the patients with concomitant IBS symptoms showed smaller improvements in severity of disease than the total dataset. Again, the small number of patients in this trial has to be taken into account.

**Table 3:** Severity of disease (CGI item 1)—change during double-blind treatment (mean ± SD, or ratio of means and 95% CI)

		Total dataset	Patients with IBS
Trial A	Menthacarin	−1.79 ± 1.27 (*n* = 19)	−1.67 ± 1.23 (*n* = 12)
	Placebo	−1.10 ± 1.33 (*n* = 20)	−1.11 ± 1.45 (*n* = 9)
	Ratio of means	1.63 [0.90, 3.68]	1.50 [0.61, 9.26]
Trial B	Menthacarin	−1.50 ± 1.25 (*n* = 108)	−1.35 ± 1.27 (*n* = 40)
	Enteric-soluble formulation	−1.40 ± 1.11 (*n* = 104)	−1.71 ± 1.22 (*n* = 42)
	Ratio of means	1.07 [0.86, 1.34]	0.79 [0.53, 1.13]
Trial C	Menthacarin	−2.10 ± 1.35 (*n* = 60)	−0.80 ± 0.84 (*n* = 5)
	Cisapride	−1.88 ± 1.40 (*n* = 58)	−1.33 ± 1.53 (*n* = 3)
	Ratio of means	1.12 [0.87, 1.45]	0.60 [−∞, +∞]

Menthacarin® is the active ingredient of the product Carmenthin® (Dr. Willmar Schwabe GmbH & Co. KG, Karlsruhe, Germany)

*CGI* clinical global impressions scale, *SD* standard deviation, *CI* confidence interval, *IBS* irritable bowel syndrome

The results for CGI items 2 (global condition change) and 3 (therapeutic effect rating) are mainly consistent with the data presented in Table 3. One minor deviation was found for item 3 in Trial C, where the therapeutic effect in the IBS subset was slightly better for Menthacarin than for cisapride (mean ± SD: 2.20 ± 1.10 vs. 2.33 ± 1.15; smaller numbers indicate more favorable ratings).

### Safety

A subset safety analysis was not performed. In patients treated with Menthacarin, the only adverse events in the five identified double-blind RCTs that were assessed to be potentially attributable to the investigational drug were isolated cases of eructation and nausea. In Trial B these effects were more common with the enteric-soluble formulation of the two essential oils than with Menthacarin, which was given as enteric-coated capsules. In none of the five trials did serious adverse events occur.

## Discussion

This subgroup analysis of randomized controlled trials indicates that Menthacarin is capable to reduce concomitant IBS symptoms of FD patients. Since the IBS subgroup improved to an extent comparable to the ITT analysis dataset with its large percentage of patients suffering from FD alone, we conclude that the symptoms associated with IBS were reduced together with the improvement of FD. Considering the large degree of symptom overlap, this result is not unexpected.

With regard to the primary outcome measure for treatment efficacy, the reduction of disease-related pain, the subgroup analysis of the data reported by May and colleagues [17] shows that the pain reduction under Menthacarin in the IBS subset was about twice as large as in the placebo group. A similar improvement was shown for the IBS-associated symptoms flatulence, diarrhea, and feelings of pressure, heaviness, or fullness in the Menthacarin group compared to the placebo group. In the data reported by Freise and Köhler [13] and by Madisch and colleagues [15], the differences in changes of intensity of pain between Menthacarin on the one hand and the enteric-soluble formulation of the fixed peppermint oil/caraway oil combination and cisapride on the other hand were negligible in patients with IBS as in the complete ITT analysis dataset. Furthermore, for the IBS subgroups, the improvement in intensity of pain was comparable for those treated with Menthacarin in all three trials. At the same time, this improvement was similar to the improvement of the patients with FD treated by Menthacarin. The trials thus are a hint that patients with IBS may benefit from treatment with Menthacarin to the same extent as patients with FD.

Medications based on herbal substances have been used in many countries for the treatment of patients with functional gastrointestinal diseases. Caraway oil exerts cholagogic and choleretic effects [20], and inhibits the smooth muscle contraction [21]. Peppermint oil as well as one of its major constituents, menthol, possess calcium antagonistic properties comparable to those of potent calcium channel blockers like verapamil, nifedipine, and diltiazem [22], by virtue of reducing calcium influx [23–25]. In addition, peppermint oil influences the transport activity of the enterocytes in the intestinal lumen by inhibiting their glucose uptake [26]. These findings explain the relaxant effect of the drug on gastrointestinal smooth muscles, which was found in both animal and human colon [25, 27], and which may contribute to its beneficial effects in IBS. Recently, several reviews and meta-analyses have been published that support the efficacy of peppermint oil in the symptomatic treatment of IBS [28–32]. Compared to placebo, the largest beneficial effects of the herbal drug were observed for abdominal pain and distension, global improvement, and quality of life. These findings explain the relaxant effect of the drug on gastrointestinal smooth muscle, which was found in both animal and human colon tissue [25, 33] and which may underlie the beneficial effects of peppermint oil in IBS, notably the reduction of abdominal pain and distension, that have been confirmed by several reviews and meta-analyses [28–32].

In functional gastrointestinal disorders, peppermint oil and caraway oil can therefore be expected to show a synergistic effect. Indeed, Micklefield and colleagues [34] have demonstrated in healthy volunteers that Menthacarin causes smooth muscle relaxation in the gastroduodenal tract by decreasing the number and amplitude of contractions in the migrating motor complex.

In our analysis, the fact that the observer ratings of severity of illness in Trial C were on average less favorable in the IBS subset than in the complete ITT analysis dataset was not reflected in the patients’ self-assessment of the severity of FD-related symptoms, and may thus indicate a certain bias introduced by the small sample size of the IBS subset in this particular trial.

The efficacy of Menthacarin in functional dyspepsia (FD) was investigated in five double-blind, randomized clinical trials: four trials demonstrated that the herbal combination is more effective than placebo or as effective as the prokinetic agent cisapride in reducing FD-associated pain and other cardinal symptoms of the disease [14–17]; in another trial, enteric-coated capsules of Menthacarin were equivalent in efficacy to an immediate-release formulation of the same essential oils while showing a better tolerability [13].

The investigators’ diagnosis of IBS in about 30% of the patients included into Trials A through C is consistent with the observations of Talley [11], who noticed that one third of the patients with FD also suffer from IBS. Considering this large subgroup within FD patients, it is particularly important that the beneficial effect of Menthacarin demonstrated in the primary publications [13–17] applied to the “typical” IBS-associated symptoms as well.

Recent meta-analyses have shown that average placebo response rates around 40% and rates of up to 70% in individual trials have to be expected in IBS [35, 36]. With roughly 45% of the patients improved in the placebo group of May et al. [17] as well as in the IBS subgroup from the same trial, our findings are consistent with the published literature on therapeutic clinical trials in IBS. The data also indicate that the percentage of patients in whom FD can be expected to improve without specific, pharmacologically active treatment does not differ between patients with or without accompanying IBS. Furthermore, these findings underline the importance of a successful physician–patient relationship that is of particular relevance in diseases that may have a strong psychological or psychophysiological component.

The subgroup analysis did not indicate any treatment-emergent risks that were specific to patients with IBS. The findings of Freise and Köhler [13] that the enteric-coated formulation leads to fewer side effects is consistent with published literature according to which adverse reactions like heartburn and eructation occur less frequently when the ethereal oil is not released in the stomach but in the bowel [37, 38]. Since the enteric-coated and the enteric-soluble formulations were comparably effective, the enteric-coated formulation may be preferable.

In conclusion, our subgroup analysis indicates that Menthacarin may offer promising perspectives for the treatment of IBS. These encouraging results merit validation in studies specifically investigating the efficacy and tolerability of Menthacarin in IBS.

## Footnotes

1 Menthacarin® is a proprietary combination of essential oils of a specified quality from Mentha x piperita L. (90 mg Peppermint oil WS® 1340) and Carum carvi (50 mg Caraway oil WS® 1520).
